# Cannabinoid CB2 Receptors Contribute to Upregulation of β-endorphin in Inflamed Skin Tissues by Electroacupuncture

**DOI:** 10.1186/1744-8069-7-98

**Published:** 2011-12-19

**Authors:** Tang-feng Su, Ling-hong Zhang, Miao Peng, Cai-hua Wu, Wen Pan, Bo Tian, Jing Shi, Hui-lin Pan, Man Li

**Affiliations:** 1 Department of Neurobiology, Tongji Medical College of Huazhong University of Science and Technology, 13 Hangkong Road, Wuhan, 430030, PR China; 2 Department of Anesthesiology and Perioperative Medicine, The University of Texas MD Anderson Cancer Center, 1515 Holcombe Boulevard, Houston, TX77030, USA

**Keywords:** acupuncture, inflammatory pain, β-endorphin, cannabinoid CB2 receptors, μ-opioid receptors

## Abstract

**Background:**

Electroacupuncture (EA) can produce analgesia by increasing the β-endorphin level and activation of peripheral μ-opioid receptors in inflamed tissues. Endogenous cannabinoids and peripheral cannabinoid CB2 receptors (CB2Rs) are also involved in the antinociceptive effect of EA on inflammatory pain. However, little is known about how peripheral CB2Rs interact with the endogenous opioid system at the inflammatory site and how this interaction contributes to the antinociceptive effect of EA on inflammatory pain. In this study, we determined the role of peripheral CB2Rs in the effects of EA on the expression of β-endorphin in inflamed skin tissues and inflammatory pain.

**Results:**

Inflammatory pain was induced by injection of complete Freund's adjuvant into the left hindpaw of rats. Thermal hyperalgesia was tested with a radiant heat stimulus, and mechanical allodynia was quantified using von Frey filaments. The mRNA level of POMC and protein level of β-endorphin were quantified by real-time PCR and Western blotting, respectively. The β-endorphin-containing keratinocytes and immune cells in the inflamed skin tissues were detected by double-immunofluorescence labeling. The CB2R agonist AM1241 or EA significantly reduced thermal hyperalgesia and mechanical allodynia, whereas the selective μ-opioid receptor antagonist β-funaltrexamine significantly attenuated the antinociceptive effect produced by them. AM1241 or EA significantly increased the mRNA level of POMC and the protein level of β-endorphin in inflamed skin tissues, and these effects were significantly attenuated by pretreatment with the CB2R antagonist AM630. AM1241 or EA also significantly increased the percentage of β-endorphin-immunoreactive keratinocytes, macrophages, and T-lymphocytes in inflamed skin tissues, and these effects were blocked by AM630.

**Conclusions:**

EA and CB2R stimulation reduce inflammatory pain through activation of μ-opioid receptors. EA increases endogenous opioid expression in keratinocytes and infiltrating immune cells at the inflammatory site through CB2R activation.

## Background

Previous studies have shown that electroacupuncture produces analgesia through increasing the β-endorphin level and activating peripheral μ-opioid receptors in the inflamed tissues [1-3]. We have shown that endogenous cannabinoids and peripheral cannabinoid CB2 receptors (CB2Rs) are also involved in the antinociceptive effect of EA on inflammatory pain [4]. Moreover, EA potentiates the CB2R expression on keratinocytes, infiltrating macrophages and T-lymphocytes in inflamed skin tissues [5], which might synthesize and release endogenous opioid peptides to reduce inflammatory pain [6-8]. However, little is known about how peripheral CB2Rs interact with the endogenous opioid system at the inflammatory site and how this interaction contributes to the antinociceptive effect of EA on inflammatory pain.

Therefore, in this study, we used a rat model of inflammatory pain to test the hypothesis that EA reduces inflammatory pain by increasing the level of β-endorphin expressed in keratinocytes and infiltrating immune cells in inflamed tissues and peripheral μ-opioid receptor activation through peripheral CB2R stimulation. To this end, we first determined the effects of the CB2R agonist AM1241 and EA on inflammatory pain and the role of peripheral μ-opioid receptors in the analgesic effect of AM1241 and EA. We then determined the role of CB2Rs in the effects of EA on the mRNA level of POMC and the protein level of β-endorphin in the inflamed skin tissues and on the percentage of β-endorphin-containing keratinocytes and infiltrating immune cells. Our findings provided new evidence that the interaction between peripheral CB2Rs and endogenous opioids contributes importantly to the analgesic effect of EA on inflammatory pain.

## Results

### Peripheral μ-opioid receptors contribute to the analgesic effects of AM1241 and EA on inflammatory pain

The purpose of the following experiments is to determine whether peripheral μ-opioid receptors contribute to the analgesic effect of the CB2R agonist AM1241 or EA on inflammatory pain. First, we examined the antinociceptive effects of AM1241 (using the AM1241 vehicle group as the control) and EA (using the sham EA group as the control) on allodynia and hyperalgesia induced by complete Freund's adjuvant (CFA) injection. Then, we determined whether pretreatment with the μ-opioid receptor antagonist β-funaltrexamine (β-FNA) attenuates the antinociceptive effect of AM1241 or EA.

**Figure 1:**
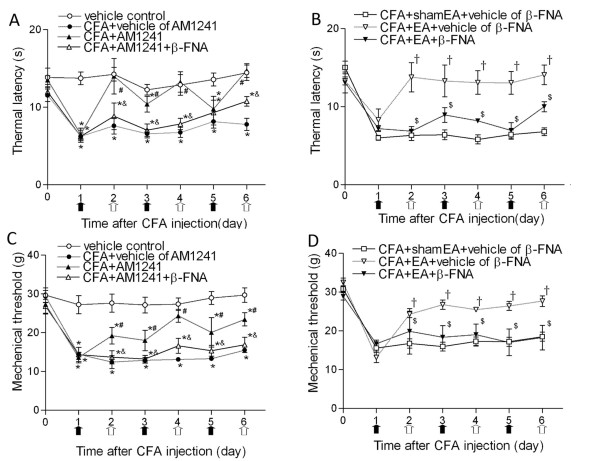
**Time course of the effects of β-FNA on the antinociceptive actions of AM1241 and EA on thermal hyperalgesia and mechanical allodynia in rats**. A, Effects of β-FNA on the AM1241 action on thermal withdrawal latency in response to heat stimulus applied to the inflamed paw. B, Effects of β-FNA on the analgesic effect of EA on thermal withdrawal latency in response to heat stimulus applied to the inflamed paw. C, Effects of β-FNA on analgesic action of AM1241 on mechanical withdrawal threshold in response to von Frey filaments applied to the inflamed paw. D, Effects of β-FNA on the analgesic action of EA on mechanical withdrawal threshold in response to von Frey filaments applied to the inflamed paw. Time 0 represents baseline values before CFA injection. EA (2 Hz) or sham EA was administered every other day for 30 min, and AM1241 or its vehicle (50 μL) was injected subcutaneously into the dorsal surface of the left hindpaw of rats at the same time of EA, as indicated by white arrows. β-FNA or its vehicle was injected 24 hours before AM1241, EA or sham EA treatment as indicated by black arrows. Data are presented as means ± SEM (n = 8 rats in each group). *P < 0.05, compared with the vehicle control group; #P < 0.05, compared with the CFA+vehicle of AM1241 group; &P < 0.05, compared with the CFA+AM1241 group; †P < 0.05, compared with the CFA+sham EA+vehicle of β-FNA group; $P < 0.05, compared with the CFA+EA+vehicle of β-FNA group (Two-way ANOVA followed by Bonferroni's test).

The baseline withdrawal thresholds in all the experimental groups were similar. CFA injection elicited typical inflammatory responses, including redness, edema, and hypersensitivities to noxious stimuli in the injected paw. These symptoms lasted for about 2 weeks as described before [9]. A large decrease in the thermal withdrawal latency and mechanical threshold was observed 1 day after CFA injection (Figure 1A-D). Compared with the vehicle group, AM1241 treatment (1 mg/kg) from days 2 to 6 significantly increased the thermal withdrawal latency and mechanical threshold in the inflamed hindpaw (Figure 1A, C). Pretreatment with β-FNA (250 μg/kg) on days 1, 3 and 5 significantly attenuated the antinociceptive effect of AM1241 (Figure 1A, C).

EA was applied to GB30 and GB34 for 30 min on days 2, 4 and 6 after CFA injection. EA significantly increased the thermal withdrawal latency and mechanical threshold, compared with those in the sham EA group (Figure 1B, D). Furthermore, pretreatment with β-FNA in the same hindpaw significantly reduced the effect of EA on thermal hyperalgesia and mechanical allodynia (Figure 1B, D).

### CB2Rs contribute to the potentiating effect of EA on the mRNA level of POMC and protein level of β-endorphin in inflamed skin tissues

The objective of the following experiments was to determine (1) the effects of AM1241 (using the AM1241 vehicle group as the control) and EA (using the sham EA group as the control) on the POMC mRNA and β-endorphin protein levels in inflamed tissues; and (2) whether blocking CB2Rs attenuates the potentiating effects of AM1241 or EA (using the AM630 vehicle group as the control) on the POMC mRNA and β-endorphin protein levels in inflamed tissues.

**Figure 2:**
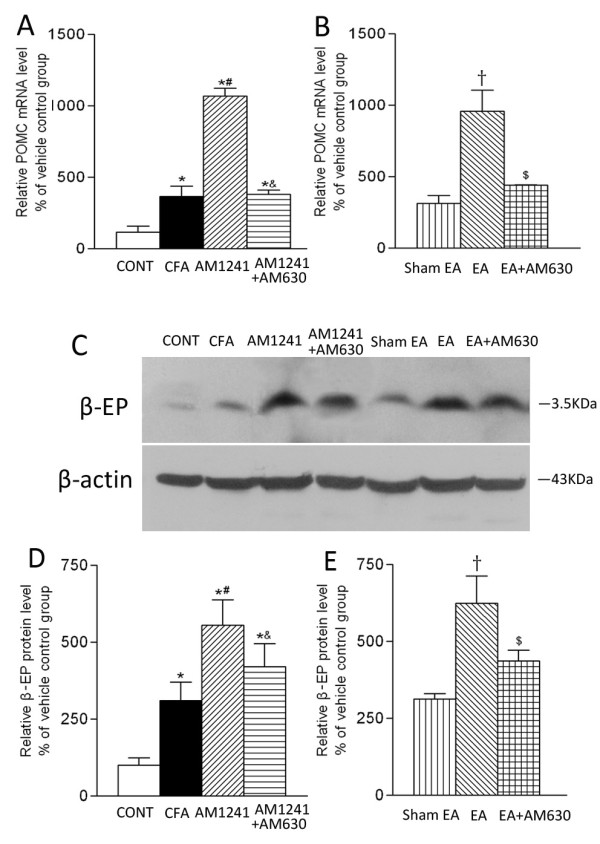
**Effects of AM1241, EA, AM1241 plus AM630, and EA plus AM630 on the mRNA level of POMC (A, B) and the protein level of β-endorphin (C, D, E) in inflamed skin tissues**. A, summary data show the relative mRNA level of POMC in the skin tissues obtained from the vehicle control (CONT), CFA+vehicle of AM1241 (CFA), CFA+AM1241 (AM1241), and CFA+AM1241+AM630 (AM1241+AM630) groups. B, summary data show the relative mRNA level of POMC in the skin tissues obtained from the CFA+sham EA+vehicle of AM630 (sham EA), CFA+EA+vehicle of AM630 (EA), and CFA+EA+AM630 (EA+AM630) groups. C, a representative gel image showing the protein level of β-endorphin in the skin tissues obtained from 7 groups of rats. β-actin was used as a loading control. The protein band at 3.5 kDa corresponds to the β-endorphin protein. D-E, summary data show the % increase in the β-endorphin protein level by AM1241 and EA with and without AM630. Data are expressed as means ± SEM (n = 6 rats in each group). * P < 0.05, compared with the vehicle control group; # P < 0.05, compared with the CFA+vehicle of AM1241 group; & P < 0.05, compared with the CFA+AM1241 group; †P < 0.05, compared with the CFA+sham EA+vehicle of AM630 group; $ P < 0.05, compared with the CFA+EA+vehicle of AM630 group (One-way ANOVA followed by Tukey's test).

The endogenous ligand of the opioid peptide β-endorphin is derived from the precursor proopiomelanocortin (POMC). The POMC mRNA was detected in the skin tissues of all groups. CFA injection increased the mRNA level of POMC in inflamed skin tissues compared with that in vehicle-injected rats (Figure 2A). AM1241 or EA significantly increased the mRNA level of POMC in inflamed skin tissues compared with that in the vehicle or sham EA group (Figure 2A, B). Pretreatment with AM630 significantly reversed the effects of AM1241 and EA on the mRNA level of POMC in inflamed tissues (Figure 2A, B).

The specific β-endorphin protein band (3.5 kDa) was present in the skin tissues samples obtained from all of the seven groups (Figure 2C). CFA injection increased the protein level of β-endorphin in the skin tissues compared with that in the CFA vehicle group (Figure 2D). Compared with the vehicle group, AM1241 treatment significantly increased the protein level of β-endorphin in inflamed tissues (Figure 2D). Also, the protein level of β-endorphin in inflamed tissues was significantly higher in the EA group than in the sham EA group (Figure 2E). In addition, pretreatment with AM630 in the same hindpaw significantly attenuated the effects of AM1241 and EA on the protein level of β-endorphin in the inflamed skin tissues (Figure 2D, E).

### CB2R activation and EA have no significant effect on the mRNA and protein levels of μ-opioid receptor-1 in inflamed skin tissues

**Figure 3:**
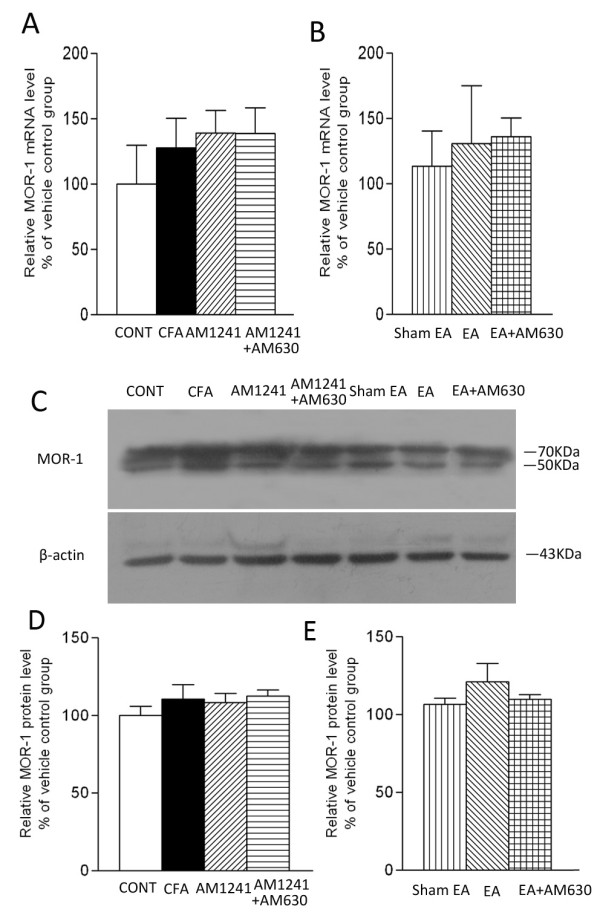
**Effects of AM1241, EA, AM1241 plus AM630, and EA plus AM630 on the mRNA (A, B) and the protein (C, D, E) levels of μ-opioid receptor-1 in inflamed skin tissues**. A, summary data show the relative mRNA level of μ-opioid receptor-1 in the skin tissues obtained from the vehicle control (CONT), CFA+vehicle of AM1241 (CFA), CFA+AM1241 (AM1241) and CFA+AM1241+AM630 (AM1241+AM630) groups. B, summary data show the relative mRNA level of μ-opioid receptor-1 in the skin tissues obtained from the CFA+sham EA+vehicle of AM630 (sham EA), CFA+EA+vehicle of AM630 (EA), and CFA+EA+AM630 (EA+AM630) groups. C, a representative gel image showing the protein level of μ-opioid receptor-1 in the skin tissues obtained from those 7 groups. β-actin was used as a loading control. Both two bands correspond to the μ-opioid receptor-1 protein. D-E, summary data show the % increase in the μ-opioid receptor-1 protein level by AM1241 and EA with and without AM630. Data are expressed as means ± SEM (n = 6 rats in each group). * P < 0.05, compared with the vehicle control group; # P < 0.05, compared with the CFA+vehicle of AM1241 group; & P < 0.05, compared with the CFA+AM1241 group; †P < 0.05, compared with the CFA+sham EA+vehicle of AM630 group; $ P < 0.05, compared with the CFA+EA+vehicle of AM630 group (One-way ANOVA followed by Tukey's test).

To determine whether CB2R stimulation or EA treatment has any effect on the μ-opioid receptor expression at the inflammatory site, we measured the mRNA and protein amount of μ-opioid receptor-1 in inflamed tissues. Treatment with either AM1241 or EA had no significant effect on the mRNA and protein levels of μ-opioid receptor-1 in inflamed skin tissues on day 6 after CFA injection compared with those in the vehicle group or sham EA group (Figure 3, A-E).

### CB2Rs contribute to the potentiating effect of EA on the level of β-endorphin expressed in keratinocytes of inflamed skin tissues

**Figure 4:**
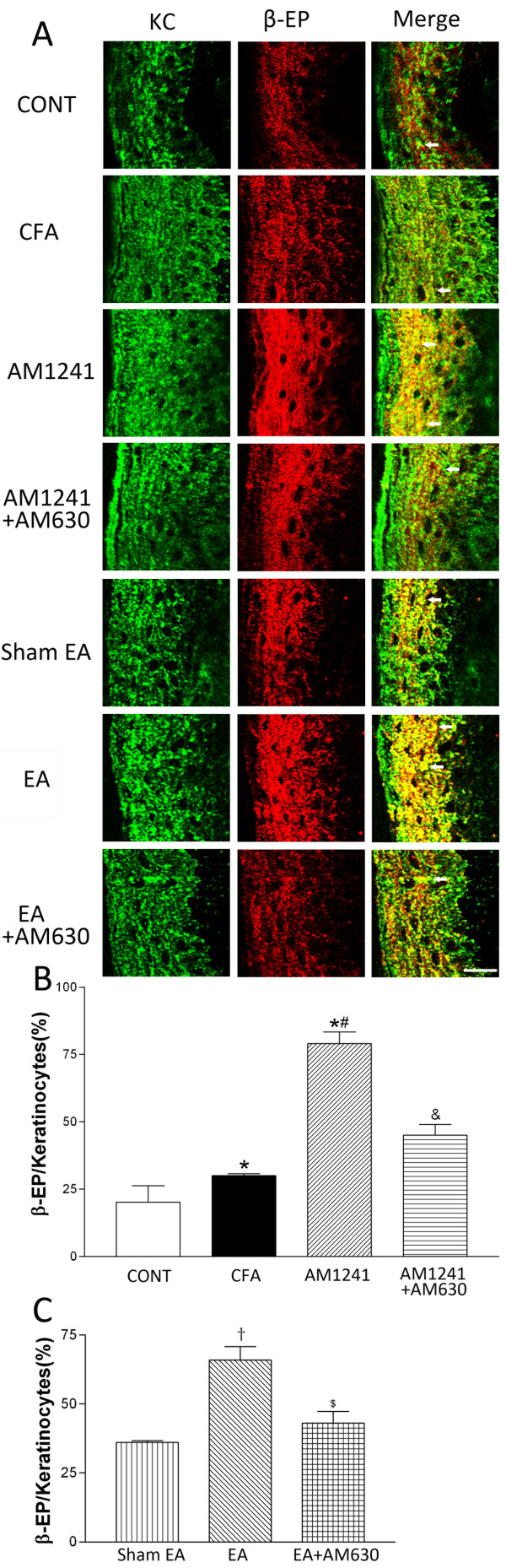
**Effects of AM1241, EA, AM1241 plus AM630, and EA plus AM630 on keratinocytes immunorecative to β-endorphin in the skin tissues**. A, representative confocal images showing pan cytokeratin positive-keratinocytes (KC, green, left panels) and β-endorphin-immunoreactive cells (β-EP, red, middle panels). The right panels are overlay images showing double-labeled β-endorphin and keratinocytes (Merge, yellow, white arrows). Images include vehicle control (CONT), CFA+vehicle of AM1241 (CFA), CFA+AM1241 (AM1241), CFA+AM1241+AM630 (AM1241+AM630), CFA+sham EA+vehicle of AM630 (sham EA), CFA+EA+vehicle of AM630 (EA), and CFA+EA+AM630 (EA+AM630) groups (Scale bar, 50 μm). B-C, summary graphs show the percentage of keratinocytes labeled with β-endorphin (β-EP) in the total of pan cytokeratin-positive cells in the skin tissues. Data are expressed as means ± SEM (n = 6 rats in each group). * P < 0.05, compared with the vehicle control group; # P < 0.05, compared with the CFA+vehicle of AM1241 group; & P < 0.05, compared with the CFA+AM1241 group; †P < 0.05, compared with the CFA+sham EA+vehicle of AM630 group; $ P < 0.05, compared with the CFA+EA+vehicle of AM630 group (One-way ANOVA followed by Tukey's test).

The epidermal layer of the skin tissues obtained from CFA-injected rats became much thicker than that in the vehicle-injected rats (Figure 4A). The β-endorphin immunoreactivity was present in keratinocytes located in the uppermost layer of the epidermis, including the stratum granulosum and the stratum spinosum in the vehicle control group. Intense β-endorphin-immunoreactivity was detected throughout the stratum granulosum and into the stratum spinosum in inflamed skin tissues (Figure 4A). The percentage of β-endorphin-immunoreactive keratinocytes was significantly increased in the skin tissues in CFA-injected rats than that in vehicle-injected rats (P < 0.05, Figure 4B).

Treatment with AM1241 or EA significantly increased the percentage of keratinocytes labeled with β-endorphin compared with that in the vehicle group or sham EA group (P < 0.05, Figure 4B, C). Furthermore, pretreatment with AM630 in the same hindpaw significantly attenuated the effect of AM1241 or EA treatment on the percentage of keratinocytes immunoreactive to β-endorphin in the inflamed skin tissues (P < 0.05, Figure 4B, C).

### CB2Rs contribute to the potentiating effect of EA on the level of β-endorphin expressed in macrophages of inflamed skin tissues

**Figure 5:**
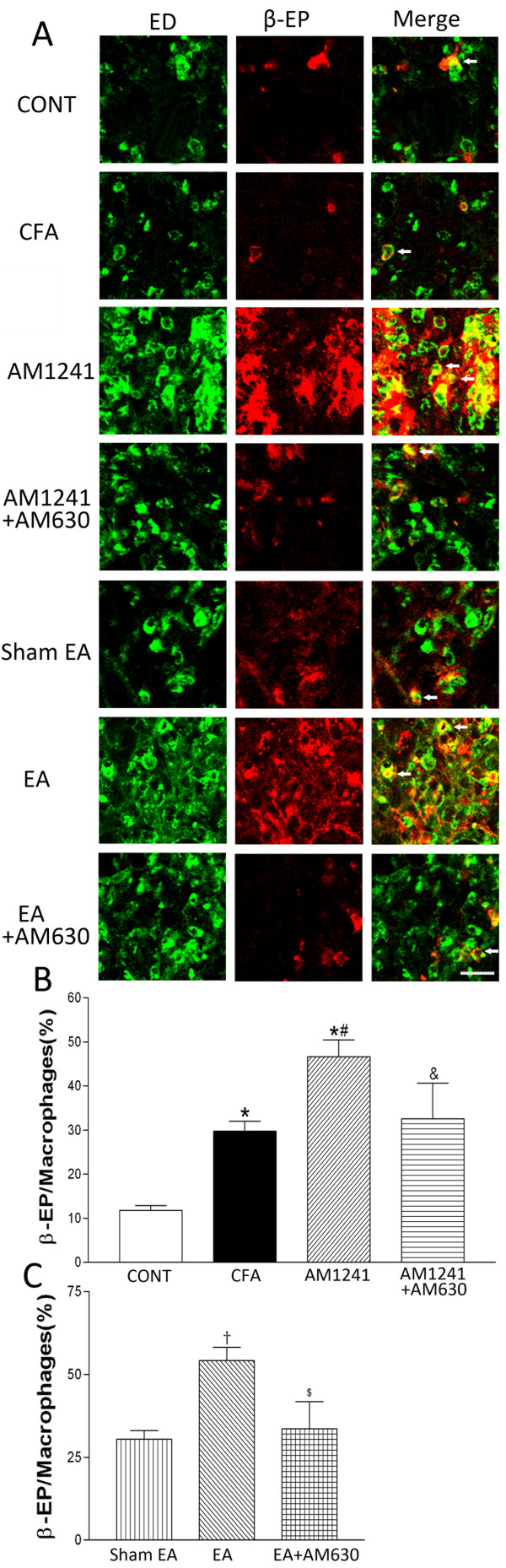
**Effects of AM1241, EA, AM1241 plus AM630, and EA plus AM630 on macrophages labeled with β-endorphin in the skin tissues**. A, representative confocal images showing ED1-positive macrophages (ED, green, left panels) and β-endorphin-immunoreactive cells (β-EP, red, middle panels). The right panels are overlay images showing double-labeled β-endorphin and macrophages (Merge, yellow, white arrows). Images include vehicle control (CONT), CFA+vehicle of AM1241 (CFA), CFA+AM1241 (AM1241), CFA+AM1241+AM630 (AM1241+AM630), CFA+sham EA+vehicle of AM630 (sham EA), CFA+EA+vehicle of AM630 (EA), and CFA+EA+AM630 (EA+AM630) groups (Scale bar, 50 μm). B-C, summary graphs show the percentage of macrophages labeled with β-endorphin (β-EP) in the total of ED1-positive macrophages in the skin tissues. Data are expressed as means ± SEM (n = 6 rats in each group). * P < 0.05, compared with the vehicle control group; # P < 0.05, compared with the CFA+vehicle of AM1241 group; & P < 0.05, compared with the CFA+AM1241 group; †P < 0.05, compared with the CFA+sham EA+vehicle of AM630 group; $ P < 0.05, compared with the CFA+EA+vehicle of AM630 group (One-way ANOVA followed by Tukey's test).

In vehicle-injected rats, only a few ED1-positive macrophages were found in the dermis of the skin, and the sizes of these cells were much smaller than those in the CFA-treated group. Also, ED1-positive macrophages were rarely labeled with β-endorphin in the skin tissues of vehicle-injected rats. In contrast, there were large numbers of infiltrating macrophages in the inflamed skin of CFA-injected rats. These cells were generally large in sizes and multivacuolated, and many of them were immunoreactive to β-endorphin (Figure 5A). The percentage of macrophages labeled with β-endorphin was also significantly increased in the inflamed skin tissues than that in vehicle-injected rats (P < 0.05, Figure 5B).

AM1241 or EA treatment significantly increased the percentage of macrophages labeled with β-endorphin in the inflamed skin tissues compared with the vehicle group or sham EA group (P < 0.05, Figure 5B, C). Furthermore, pretreatment with AM630 in the same hindpaw significantly blocked the effects of AM1241 and EA on the percentage of macrophages immunoreactive to β-endorphin in the inflamed skin tissues (P < 0.05, Figure 5B, C).

### CB2Rs contribute to the potentiating effect of EA on the level of β-endorphin expressed in T-lymphocytes of inflamed skin tissues

**Figure 6:**
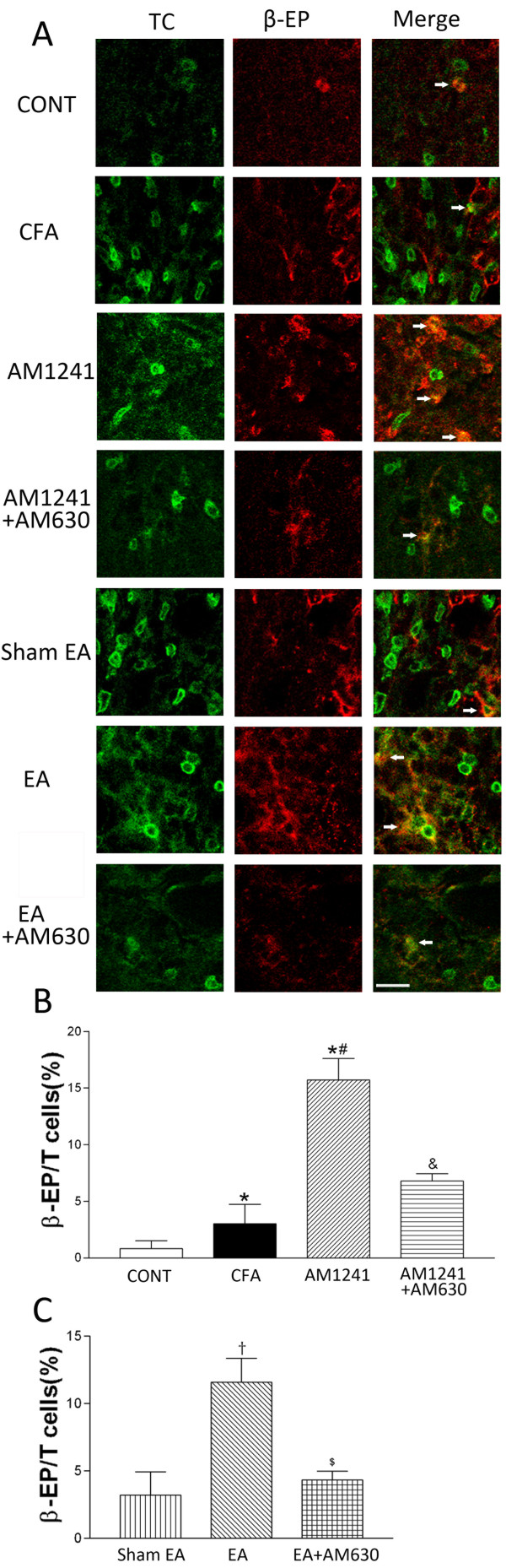
**Effects of AM1241, EA, AM1241 plus AM630, and EA plus AM630 on T-lymphocytes with β-endorphin immunorecativity in the skin tissues**. A, representative confocal images show TCR-positive T-lymphocytes (TC, green, left panels) and β-endorphin-immunoreactive cells (β-EP, red, middle panels). The right panels are overlay images showing T-lymphocytes labeled with β-endorphin (Merge, yellow, white arrows). Images include vehicle control (CONT), CFA+vehicle of AM1241 (CFA), CFA+AM1241 (AM1241), CFA+AM1241+AM630 (AM1241+AM630), CFA+sham EA+vehicle of AM630 (sham EA), CFA+EA+vehicle of AM630 (EA), and CFA+EA+AM630 (EA+AM630) groups (Scale bar, 50 μm). B-C, summary graphs show the percentage of double-labeled T-lymphocytes labeled with β-endorphin (β-EP) in the total of TCR-positive T-lymphocytes in the skin tissues. Data are expressed as means ± SEM (n = 6 rats in each group). * P < 0.05, compared with the vehicle control group; # P < 0.05, compared with the CFA+vehicle of AM1241 group; & P < 0.05, compared with the CFA+AM1241 group; †P < 0.05, compared with the CFA+sham EA+vehicle of AM630 group; $ P < 0.05, compared with the CFA+EA+vehicle of AM630 group (One-way ANOVA followed by Tukey's test).

TCR-positive T-lymphocytes were present in the dermis of the skin sections obtained from CFA-injected rats and had the size and oval shape consistent with the typical characteristics of T-lymphocytes [10]. However, these cells were rarely present in the rat skin tissues in the vehicle-injected rats, and only few of them were immunoreactive to β-endorphin. In the skin tissues from CFA-treated rats, there was a marked increase in infiltrating TCR-positive T-lymphocytes and some of them were labeled with β-endorphin (Figure 6A). Also, the percentage of T-lymphocytes labeled with β-endorphin in the total of T-lymphocytes was significantly increased in the inflamed skin tissues compared with that in the control skin tissues (P < 0.05, Figure 6B).

Treatment with AM1241 or EA significantly increased the percentage of T-lymphocytes immunoreactive to β-endorphin in the inflamed skin tissues compared with that in the vehicle group or sham EA group (P < 0.05, Figure 6B, C). Pretreatment with AM630 in the same hindpaw significantly reversed the effect of AM1241 or EA treatment on the percentage of T-lymphocytes labeled with β-endorphin in the inflamed skin tissues (P < 0.05, Figure 6B, C).

## Discussion

It has been reported that endogenous opioid peptides released from immune cells can act on opioid receptors on peripheral sensory nerves to reduce inflammatory pain [11]. Endogenous opioid peptides have been found in increased levels in the inflamed tissue with β-endorphin being the most abundant [6,7,12]. The μ-opioid receptors are distributed on the peripheral sensory nerves and their terminals [13], and the μ-opioid receptor-positive nerve fibers in the subcutaneous tissue are significantly increased after tissue inflammation [14]. It has been reported that EA increases the content of β-endorphin in the inflamed tissues [1]. The analgesic effect of EA on pain caused by carrageenan injection is dose-dependently antagonized by intraplantar (i.pl.) injection, but not by intraperitoneal (i.p.) or intravenous (i.v.) administration of naloxone or selective μ-opioid receptor antagonists [2,3]. Although both CB1Rs and CB2Rs are found in peripheral tissues [15,16], activation of peripheral CB2Rs produces a particularly potent analgesic effect on inflammatory pain [17,18]. We have shown that the antinociceptive effect of EA on inflammatory pain is blocked by CB2R, but not CB1R, antagonists [4]. Furthermore, EA treatment significantly potentiates the anandamide concentration in inflamed skin tissues [4] and increases the number of CB2R-positive keratinocytes, macrophages, and T-lymphocytes at the site of tissue inflammation [5]. In the present study, we found that β-FNA, a selective and irreversible μ-opioid receptor antagonist [19], reversed the inhibitory effect of EA or the CB2R agonist AM1241 on thermal hyperalgesia and mechanical allodynia caused by CFA. Thus, our results suggested that peripheral μ-opioid receptors contribute to the analgesic effects of AM1241 and EA in inflammatory pain conditions.

The most salient finding of our present study is that the CB2R agonist AM1241 and EA significantly increased the mRNA level of POMC and protein level of β-endorphin in the inflamed skin tissues. The potentiating effects of AM1241 and EA on POMC and β-endorphin expression were largely blocked by local administration of the CB2R antagonist AM630. Thus, our data suggested that peripheral CB2Rs contribute to the potentiating effects of AM1241 and EA on the expression level of POMC and β-endorphin at the site of inflammation. POMC-related opioid peptides have been found in leukocytes of many vertebrates and invertebrates [20]. POMC transcripts are upregulated in lymphocytes from rats with paw inflammation [21], and the enzymes (e.g., prohormone convertases and carboxypeptidase) required for proteolytic processing of POMC are expressed in leukocytes [22]. Another type of cells that might contain endogenous opioids is keratinocytes, which synthesize POMC and its derivatives β-LPH and β-endorphin [23,24]. It has been reported that AM1241 stimulates β-endorphin release from the paw glabrous skin and cultured human keratinocytes (HaCaT) cells [25]. Naloxone can reverse the antinociceptive effects produced by local injection of AM1241 [25]. CB2R activation by AM1241 and EA may stimulate the expression level and release of β-endorphin, which then acts on peripheral μ-opioid receptors to inhibit nociception [26]. It remains unclear how CB2R activation leads to increased POMC and β-endorphin expression level during tissue inflammation. Activation of CB2Rs promotes the phosphorylation of p42/p44 mitogen-activated protein kinase (MAPK) [27,28], and activation of p42/p44 MAPK is linked to increased expression of the growth-related gene Krox-24 [27]. Also, activation of the CB2Rs induce upregulation of nine genes involved in cytokine synthesis, regulation of transcription, and cell differentiation; most of which are under the control of the transcription factor NF-κB [29]. It is possible that activation of CB2Rs may modulate the expression of POMC and β-endorphin through the MAP kinase signaling cascade. Our study provides novel evidence that CB2R activation by AM1241 and EA increases the β-endorphin expression level, which contributes to the analgesic effect of EA on inflammatory pain.

During inflammation, preexisting or newly synthesized μ-opioid receptors in the primary sensory neurons are transported to the inflamed tissue and the spinal dorsal horn [30]. Increased peripheral μ-opioid receptor expression can last up to 3 days after tissue inflammation [14,31], and an increase in μ-opioid receptor mRNA level can occur in the dorsal root ganglia (DRG) 1 to 2 h after CFA injection [32]. Previous studies showed that there are no changes in the mRNA and protein levels of μ-opioid receptors in the DRG or the paw tissues 7 days after CFA-induced inflammation [33]. In the present study, we found no evidence that AM1241 or EA altered the mRNA and protein levels of μ-opioid receptors in the inflamed tissues. Because EA treatment can increase the level of endogenous anandmide [4,5] and the level of β-endorphin in the inflamed skin tissues, activation of CB2Rs could amplify the analgesic effect of EA by increasing the amount of endogenous opioid ligands but not the μ-opioid receptor expression level. Our findings highlight the important role of interactions between peripheral CB2Rs and the endogenous opioid system in the antinociceptive effect of EA on inflammatory pain.

We further identified the cell types involved in increased β-endorphin level in the inflamed skin tissues by AM1241 and EA treatment. Immune cells (e.g., macrophages and T-lymphocytes) recruited to the inflammatory site and keratinocytes can synthesize and release β-endorphin [6,12,21,34-36]. In the present study, we found CFA-induced inflammation significantly increased the percentage of keratinocytes, macrophages, and T-lymphocytes that are immunoreactive to β-endorphin in inflamed tissues. Interestingly, both AM1241 and EA treatments produced a further increase in the percentage of keratinocytes, macrophages, and T-lymphocytes labeled with β-endorphin in the inflamed skin tissues. EA treatment can increase the anadamide level and the percentage of keratinocytes, macrophages, and T-lymphocytes expressing CB2Rs [4,5]. Also, the antinociceptive actions of EA are mediated by local CB2Rs [4]. It has been reported that CB2R activation can increase the release of β-endorphin from keratinocytes [5]. Because EA increased the percentage of β-endorphin-containing keratinocytes, macrophages, and T-lymphocytes in inflamed tissues and the EA analgesic effect was blocked by a selective μ-opioid receptor antagonist, it is likely that EA increases the release of β-endorphin from keratinocytes, macrophages, and T-lymphocytes at the site of inflammation.

## Conclusions

In summary, we found that peripheral μ-opioid receptors contribute importantly to the antinociceptive effect produced by CB2R activation and EA in a rat model of inflammatory pain. Through activation of CB2Rs, EA significantly increases the mRNA level of POMC, the protein level of β-endorphin, and the percentage of keratinocytes, macrophages, and T-lymphocytes expressing β-endorphin in the inflamed skin tissues. Our study provided novel evidence that EA upregulates the endogenous opioid system by activating peripheral CB2Rs. This new information improved our understanding of the mechanisms underlying acupuncture analgesia. The interaction between endogenous opioid and cannabinoid systems at the inflammatory site could lead to improved treatments for inflammatory pain conditions.

## Methods

### Animal models

Experiments were carried out on male adult Sprague-Dawley rats (180-200 g) purchased from Experimental Animal Center of Tongji Medical College of Huazhong University of Science and Technology. All procedures were approved by the Animal Care Committee at Huazhong University of Science and Technology and conformed to the ethical guidelines of the International Association for the Study of Pain [37]. The rats were individually housed in cages with a 12-hr light/dark cycle and had free access to food and water.

Inflammation was induced by injecting 50 μL of complete Freund's adjuvant (CFA; Sigma, St. Louis, MO) subcutaneously into the dorsal surface of left hindpaw of rats using a 25-gauge hypodermic needle [38]. The injections were carried out under light anesthesia by means of ether inhalation. We selected the dorsal surface of the hindpaw as the injection site in order to produce an inflammatory pain focus in Gallbladder Channel of Foot Shaoyang, where GB30 and GB34 are located, according to the meridian theory of traditional Chinese medicine [4,39]. In separate rats, mineral oil was injected into the dorsal surface of the hindpaw and was used as the vehicle control.

### Electroacupuncture (EA) treatment

In the EA treatment group, the rats received EA on the ipsilateral "Huantiao" (GB30) and "Yanglingquan" (GB34) once every other day, starting at the second day after CFA injection. EA (1 mA and 0.1 ms) was administered at 2 Hz for 30 min. Current was delivered with a modified current-constant Han's Acupoint Nerve Stimulator (LH202, Huawei Co.Ltd., Beijing, China). GB30 and GB34 were chosen based on their effective use in reducing inflammatory pain in rats [4,9].

Two acupuncture needles were inserted into two acupoints corresponding to GB30 and GB34 in humans. GB30 is located at the junction of the lateral 1/3 and medial 2/3 of the distance between the greater trochanter and the hiatus of the sacrum; and GB34 lies on the lateral aspect of the leg in the depression anterior and inferior to the head of the fibula in rats [40]. During EA treatment, each rat was placed in an inverted clear plastic chamber (approximately 4 cm × 4 cm × 11 cm) but was not restrained. The animals remained still during EA treatment and showed no evident signs of distress. For sham control, acupuncture needles were inserted ipsilaterally into GB30 and GB34 without electrical stimulation or manual needle manipulation.

### Drug administration

AM1241 is a potent and selective CB2R agonist [41]. AM630 is a highly specific CB2R antagonist with a 70-165-fold selectivity for the CB2R in vitro [42]. AM1241 (1 mg/kg, Enzo Life Sciences, PA, USA) and AM630 (150 μg/kg, Enzo Life Sciences, PA, USA) were dissolved in the vehicle solution containing 5% Tween-80 and 5% DMSO in normal saline. β-FNA is a selective and irreversible μ-opioid receptor antagonist [19]. β-FNA (250 μg/kg, Sigma, St. Louis, MO, USA) was dissolved in methanol. The dose of AM1241, AM630 and β-FNA was selected based on the previous studies [4,41,43] and our preliminary dose-response study and was the lowest that produced reproducible behavioral effects. AM1241 or its vehicle (50 μL) was injected subcutaneously into the dorsal surface of the left hindpaw of rats once every other day, starting at the second day after CFA injection. AM630 or the vehicle (50 μL) was injected subcutaneously into the dorsal surface of the left hindpaw at the same injection time of AM1241 or 5 min before EA or sham EA treatment each time. β-FNA or the vehicle (50 μL) was injected subcutaneously into the dorsal surface of the left hindpaw 24 h before AM1241, EA or sham EA treatment each time. The investigators involved in behavioral tests and biochemical assays were blinded to the drug injection throughout the study.

### Nociceptive behavioral tests

The behavioral tests were performed 3 times before CFA injection and once every day, starting from the first day after CFA injection. The animals were habituated to the testing environment for 30 min. Thermal hyperalgesia was assessed by exposing the mid-plantar surface of the hindpaw to a beam of radiant heat through a transparent glass surface using a plantar analgesia meter (Ugo Basile, Italy), as previously described [44]. The withdrawal latency was recorded for both left and right hindpaws as the time taken from the onset of radiant heat stimulation to withdrawal of the hindpaw.

Mechanical allodynia was assessed by placing rats on an elevated mesh floor, and the tactile threshold was measured by using an electronic von Frey anesthesiometer (Ugo Basile, Italy) applied to the plantar surface of the left hindpaw. The force (g) needed to produce a paw withdrawal response was tested 4 times separated by 2- to 3-min intervals. A mean value of 4 consecutive measurements was used.

### Real-time PCR procedures

The skin tissues (5 mm × 5 mm × 2 mm) at the site of CFA or vehicle injection were removed on day 6 after CFA injection (i.e., 25 min after the third EA treatment or 60 min after the third AM1241 treatment). The skin tissues were excised from rats immediately after the animals were anesthetized with an overdose of sodium pentobarbitone (120 mg ⁄kg, i.p.) and decapitated. Total RNA was isolated from the skin specimens using Trizol reagent (Invitrogen, CA, USA). Aliquots of 3 μg total RNA were reverse transcribed into cDNA using ReverTra Ace-α-TM (Toyobo, Osaka, Japan). The 20 μL (total volume) of the PCR mixture consisted of 1 μL diluted cDNA, 10 μL SYBR green-PCR master mixture (2×) (Toyobo, Osaka, Japan), and 0.3 μM of each primer. Real-time PCR was performed using the Stratagene Mx3005P. Sequence-specific primers were listed in Table 1.

**Table 1:** List of primers used for real-time PCR

Gene names	primers
POMC	sense, 5'- GCAACGGAGATGAACAGCC-3'
	
	antisense, 5'- TCTTCCTCCGCACGCCTCT-3'

μ-opioid receptor-1	sense, 5'-GCATTGCTTTGGGTTACACG-3'
	
	antisense, 5'-CTGTATTAGCCGTGGAGGGA-3'

β-actin	sense, 5'-CACCCGCGAGTACAACCTTC-3'
	
	antisense, 5'-CCCATACCCACCATCACACC-3'

The expression level of each gene was determined by the threshold cycle (CT). Samples that had larger amounts of a particular gene had correspondingly lower CT values. For each sample, a CT value was obtained for POMC, μ-opioid receptor-1, and β-actin. The CT value of β-actin was subtracted from that of POMC or μ-opioid receptor-1 to obtain a ΔCT value. The mRNA amount, normalized to the endogenous control (β-actin), was given by 2^-ΔΔCt^. Results of six independent experiments were expressed as the % change over relative mRNA level of the vehicle control group.

### Western blotting

The plantar skin of the left hindpaw (5 mm × 5 mm × 2 mm) was removed as described above, minced with scissors, and homogenized in 300 μL RIPA Lysis Buffer (Beyotime Biotechnology, Nanjing, China) with 2 mM phenylmethylsulfonyl fluoride and the protease inhibitor cocktail (Roche Applied Science, CT, USA), and centrifuged at 12,000 × g for 10 min. The pellet was discarded and protein concentrations from the supernatant were determined using the Enhanced BCA Protein Assay Kit (Beyotime Biotechnology, Nanjing, China). To quantify β-endorphin protein level, 150 μg total protein of each tissue was processed with 2 × Tricine-SDS-PAGE loading buffer (Tiandz Biotech, Beijing, China), and then separated on SDS-PAGE gel as previously described [45]. To determine the μ-opioid receptor-1 protein level, 30 μg total protein of each tissue was processed with 5 × SDS-PAGE loading buffer at 95°C for 5 min, and then separated on a 12% glycine-SDS-PAGE gel. The proteins were transferred onto a PVDF membrane, blocked for 1 hr in 5% nonfat dry milk in Tris-buffered saline (TBS) containing 0.1% Tween-20. The membrane was incubated with the rabbit anti-β-endorphin antibody (1:20,000; Abcam, San Francisco, CA, USA) and rabbit anti-μ-opioid receptor-1 antibody (1:200; Santa Cruz, CA, USA) at 4°C overnight, respectively. The molecular weight of the protein band detected matched with that reported in previous studies. The specificity of the anti-β-endorphin antibody has been shown by detecting the specific β-endorphin band at 3.5 kDa [22]. Blotting using the μ-opioid receptor-1 antibody resulted in two protein bands at ~50 and ~70 kDa [46,47]. After washes in 0.1% TBS-Tween 20, the membranes were then incubated with a horseradish peroxidase-conjugated goat anti-rabbit secondary antibody (1:20000; Jackson ImmunoResearch, MD, USA) for 1 h at room temperature, and then washed three times. The enhanced chemiluminescence method (ECL Plus Western blotting detection reagents; Pierce, IL, USA) was used to reveal the protein bands according to the manufacturer's protocol. The optical density of each band was then measured with a computer-assisted imaging analysis system (Quantity one, Bio-Rad, Hemel Hempstead, UK) and normalized with β-actin. Results of six independent experiments were expressed as the % change over the protein amount in the CFA vehicle control group.

### Double-immunofluorescence labeling procedures

Rats were deeply anesthetized with an overdose of sodium pentobarbitone (120 mg/kg, i.p.) and were transcardially perfused with normal saline and 4% paraformaldehyde in 0.1 M phosphate buffer at pH 7.4. The skin tissues with the injection point in the middle was harvested. Tissues were postfixed at 4°C in the perfusion fixative for 8 h, cryoprotected in 30% sucrose overnight, and sectioned at 15 μm on a cryostat in a plane perpendicular to the skin surface and parallel to the long axis of the foot. The sections were mounted onto gelatin-coated slides, air-dried overnight, and used for double-immunofluorescence labeling of β-endorphin with markers of keratinocytes, macrophages, or T-lymphocytes [14,25].

Double immunolabeling was performed with rabbit anti-β-endorphin (1:500; Abcam, Cambridge, UK) and mouse monoclonal anti-pan cytokeratin antibody (1:100; Abcam, Cambridge, UK) for identification of keratinocytes, mouse monoclonal anti-CD68 antibody (Clone ED1, 1:200; Serotec, Oxford, UK) for detection of macrophages, or mouse monoclonal anti-αβ T cell receptor (TCR) antibody (Clone R73, 1:200; BD Biosciences-PharMingen, San Diego, CA, USA) for identification of T-lymphocytes. The specificity of the β-endorphin antibody has been demonstrated by antigen preabsorption with the corresponding blocking peptides. The anti-pan cytokeratin antibody reacts specifically with the skin epidermal keratinocytes [48]. The anti-CD68 antibody specifically labels macrophages in the tissues [49]. The anti-TCR antibody is a specific marker for peripheral T-lymphocytes and does not react with γδ TCR-bearing T cells [50].

All sections were blocked for 30 min with 5% donkey serum and 0.2% Tween-20 in PBS, followed by incubation at 37°C for 1 hr then at 4°C overnight with the primary antibody diluted in PBS containing 5% bovine serum albumin (BSA). The sections were washed 4 times with 0.05% Tween-20 in PBS for 5 min, and incubated with a mixture of secondary antibodies: donkey anti-mouse IgG conjugated with Dynight 488 (1:400; Jackson ImmunoResearch) and donkey anti-rabbit IgG conjugated with Dynight 594 (1:500; Jackson ImmunoResearch). Sections were washed 4 times with 0.05% Tween-20 in PBS for 5 min, and then treated with the fluorescence-mounting medium to inhibit quenching of fluorescence before being coverslipped. Negative controls were included by omitting the primary antibodies and with primary antibodies preabsorbed with their specific blocking peptides in the above procedures, which resulted in no positive labeling in the skin tissues. Six rats per group were used for the double-immunolabeling and cell counting.

### Imaging acquisition and analysis

Digital confocal images were acquired using a laser scanning confocal microscope (FV500-IX71, Olympus, Japan). The sections were scanned using excitation at 488 nm (argon laser) for Dylight 488 and at 594 nm (helium neon laser) for Dylight 594. A total of 3-4 sections were imaged from the same skin tissues in each rat, and counting of single- and double-labeled cells was done on confocal images randomly taken from three view fields in each section. Cell counting was performed by an investigator in a blind fashion using NIH Image J software (Bethesda, MD, USA). The percentage of double-labeled cells in the total of single-labeled cells was used for statistical analysis.

### Statistical analysis

Data are presented as means ± SEM. We used one-way ANOVA (mRNA and protein levels and the percentage of double-labeled cells) or two-way ANOVA (behavioral data) to determine the overall effect of interventions. Tukey's post hoc test was then used to determine the statistical difference in the mRNA and protein levels and the percentage of double-labeled cells between individual groups. To determine the statistical difference in the withdrawal thresholds between different groups and time points, we used Bonferroni's post hoc test. A P value of less than 0.05 was considered statistically significant.

## List of abbreviations

EA: electroacupuncture; CFA: complete Freund's adjuvant; CB2Rs: cannabinoid CB2 receptors; CB1Rs: cannabinoid CB1 receptors; POMC: proopiomelanocortin; MOR: μ-opioid receptor; β-EP: β-endorphin; β-FNA: β-funaltrexamine; MAPK: mitogen-activated protein kinase; DRG: dorsal root ganglia.

## Competing interests

The authors declare that they have no competing interests.

## Authors' contributions

TS carried out the animal model preparation, drug administration, real-time PCR and Western blotting. LZ conducted double-immunofluorescence labeling procedures. MP conducted nociceptive behavioral tests. CW conducted EA treatment. WP participated in imaging acquisition and analysis. BT helped to perform Western blotting analysis. JS helped to analyze data. HP participated in study design and performed manuscript writing. ML conceived of the study, oversaw the design, coordinated the study, and edited the manuscript. All authors read and approved the final manuscript.
